# Structural and Functional Insights into (*S*)-Ureidoglycolate Dehydrogenase, a Metabolic Branch Point Enzyme in Nitrogen Utilization

**DOI:** 10.1371/journal.pone.0052066

**Published:** 2012-12-20

**Authors:** Myung-Il Kim, Inchul Shin, Suhee Cho, Jeehyun Lee, Sangkee Rhee

**Affiliations:** 1 Department of Agricultural Biotechnology, Seoul National University, Seoul, Korea; 2 Center for Fungal Pathogenesis, Seoul National University, Seoul, Korea; Universite de Sherbrooke, Canada

## Abstract

Nitrogen metabolism is one of essential processes in living organisms. The catabolic pathways of nitrogenous compounds play a pivotal role in the storage and recovery of nitrogen. In *Escherichia coli*, two different, interconnecting metabolic routes drive nitrogen utilization through purine degradation metabolites. The enzyme (*S*)-ureidoglycolate dehydrogenase (AllD), which is a member of l-sulfolactate dehydrogenase-like family, converts (*S*)-ureidoglycolate, a key intermediate in the purine degradation pathway, to oxalurate in an NAD(P)-dependent manner. Therefore, AllD is a metabolic branch-point enzyme for nitrogen metabolism in *E. coli*. Here, we report crystal structures of AllD in its apo form, in a binary complex with NADH cofactor, and in a ternary complex with NADH and glyoxylate, a possible spontaneous degradation product of oxalurate. Structural analyses revealed that NADH in an extended conformation is bound to an NADH-binding fold with three distinct domains that differ from those of the canonical NADH-binding fold. We also characterized ligand-induced structural changes, as well as the binding mode of glyoxylate, in the active site near the NADH nicotinamide ring. Based on structural and kinetic analyses, we concluded that AllD selectively utilizes NAD^+^ as a cofactor, and further propose that His116 acts as a general catalytic base and that a hydride transfer is possible on the B-face of the nicotinamide ring of the cofactor. Other residues conserved in the active sites of this novel l-sulfolactate dehydrogenase-like family also play essential roles in catalysis.

## Introduction

Nitrogen is an essential element in living organisms. In plants, nitrogen is obtained directly from the environment; for example, nitrogen can be fixed by soil bacteria in leguminous plants [1] and can be transported as urea [2] or the heterocyclic compound allantoin in *Arabidopsis thaliana* [3]. In addition to these established processes for nitrogen uptake, many organisms efficiently utilize nitrogen by recovering it from metabolites via catabolic pathways. For example, under anaerobic conditions, *Escherichia coli* uses allantoin as an exclusive nitrogen source, suggesting that nitrogen is isolated from allantoin or its metabolites [4].

**Figure 1:**
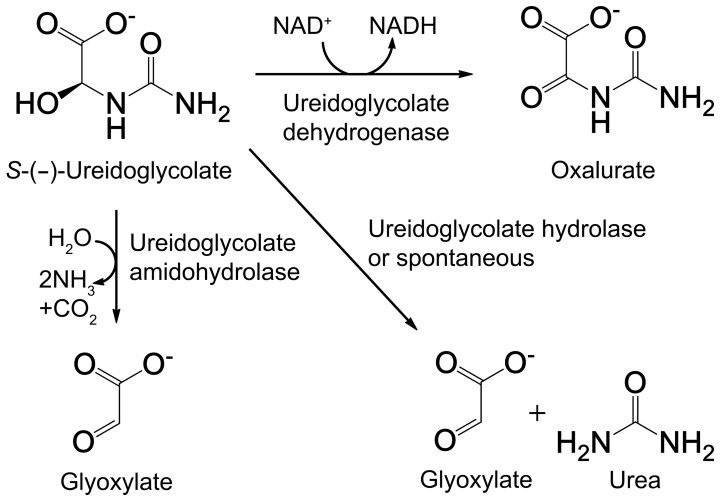
Scheme for conversion of (*S*)-ureidoglycolate via three different enzymes.

Recent comparative genomic studies [5], [6], combined with biochemical and structural analyses [7]–[14], revealed that purine molecules and allantoin, which both contain nitrogen as a major constituent, are metabolized to recover nitrogen. This purine catabolic pathway, also known as the ureide pathway, consists of two major metabolic routes linked by (*S*)-allantoin as an intermediate. The first half of the pathway catalyzes the degradation of uric acid, a major product of early stage of purine catabolism, into stereospecific (*S*)-allantoin through three-step enzyme reactions [5]. Next, (*S*)-allantoin is converted into (*S*)-ureidoglycolate via three consecutive enzymatic reactions, releasing two molecules of ammonia (Figure S1) [6]. Bioinformatic and biochemical analyses indicated that the enzymes necessary for producing (*S*)-ureidoglycolate by the ureide pathway are conserved in all plants sequenced to date, as well as in some bacteria and fungi [5], [6]. (*S*)-Ureidoglycolate can undergo three different processes depending on the distinct metabolic pathways present in different organisms (Figure 1). In plants, (*S*)-ureidoglycolate is further hydrolyzed into one molecule of glyoxylate and two molecules of ammonia [6], whereas in some bacteria (depending on oxygen and nitrogen availability), it is subject to an enzyme-dependent dehydrogenation reaction, forming oxalurate [15], [16]. Alternatively, (*S*)-ureidoglycolate can produce one molecule of glyoxylate and urea in an enzyme-dependent hydrolysis or enzyme-independent spontaneous reaction [6]. Therefore, the released ammonia and/or urea serves as a nitrogen source for downstream pathways unique to each organism.

The enzyme (*S*)-ureidoglycolate dehydrogenase (EC 1.1.1.154) was designated as the gene product of *allD* in *E. coli* [17] and belongs to a member of l-sulfolactate dehydrogenase-like protein family [18]. (*S*)-Ureidoglycolate dehydrogenase (AllD) catalyzes the formation of oxalurate from (*S*)-ureidoglycolate using an NAD(P)-dependent reaction (Figure 1) [16], and subsequently, oxalurate is converted into ammonia and CO_2_ by sequential reactions of oxamate transcarbamoylase and carbamate phosphotransferase [19]. Although AllD plays a pivotal role in *E. coli* as a metabolic branch-point enzyme in nitrogen utilization, its structural and functional features are unknown, except for a preliminary structure analysis of its apo form by the New York SGX Research Center for Structural Genomics (PDB code 1XRH). Here, we investigated the structural and functional features of *E. coli* AllD. Initial functional studies indicated that NAD^+^, not NADP^+^, serves as the preferred reaction cofactor (Figure S2). Therefore, a structure of AllD in its apo form was determined at 2.13 Å resolution, as well as a binary complex at 1.64 Å resolution with the NADH cofactor, and a ternary complex at 1.77 Å resolution with NADH and glyoxylate, a product yielded from the spontaneous degradation of oxalurate. Based on structural and functional analysis, we proposed a catalytic mechanism for AllD, as well as functional roles for the active site residues.

## Results

### Overall Structure of AllD in Apo Form

All crystals used in this study belonged to space group P*4_2_2_1_2*, with two monomers per asymmetric unit (Table 1). The structure of the apo form of AllD was solved by molecular replacement with a monomer of *E. coli* AllD (PDB code 1XRH by New York SGX Research Center for Structural Genomics) as the search model. The dimeric structure observed in the asymmetric unit represents the functional unit of AllD, consistent with size-exclusion chromatography, in which AllD eluted as a dimer.

**Table 1:** Data collection and refinement statistics.

	AllD	AllD-NADH	AllD-NADH-glyoxylate
**Soaking Condition**			
Concentration		100 mM NADH (10)	100 mM NADH (30)
(Soaking time, min)			7.5 mM oxalurate (20)
**Data collection**			
Space group	*P4_2_2_1_2*	*P4_2_2_1_2*	*P4_2_2_1_2*
Cell dimensions			
a, b, c (Å)	162.4, 162.4, 61.8	162.5, 162.5, 61.0	162.8, 162.8, 61.5
Wavelength (Å)	1.00000	0.97948	1.23984
Resolution^a^ (Å)	50–2.13	50–1.64	50–1.77
	(2.21–2.13)^b^	(1.70–1.64)	(1.83–1.77)
Total reflections	915786	1189325	1265226
Unique reflections	46352	99377	80488
Redundancy	19.8 (12.5)	12.0 (12.1)	15.7 (15.0)
*R*_sym_ or *R*_merge_ c (%)	15.9 (99.6)	9.7 (96.8)	7.4 (58.3)
I/σI	14.6 (2.1)	28.3 (2.1)	46.7 (4.6)
Completeness (%)	99.1 (94.9)	99.5 (100.0)	99.7 (97.3)
Wilson B-factor (Å^2^)	21.3	21.7	21.3
**Refinement**			
Resolution (Å)	50–2.13	50–1.64	50–1.77
*R*_work_ d/*R*_free_ e	22.2/24.0	23.0/24.6	22.9/24.8
No. atoms			
Protein^f^	4959	4951	4951
NADH		88	88
Glyoxylate			5
Water	502	711	707
Average *B*-factors (Å^2^)			
Protein	34.3	33.9	29.9
NADH		25.6	25.0
Glyoxylate			39.2
Water	32.8	36.1	33.2
R.m.s deviations			
Bond lengths (Å)	0.009	0.007	0.007
Bond angles (°)	1.113	1.139	1.141
Ramachandran plot			
Favored (%)	95.37	97.21	97.21
Allowed (%)	4.17	2.48	2.17
Outliers^g^ (%)	0.46	0.31	0.62

a High-resolution cutoff was based on the CC_1/2_ value obtained from the program Aimless in CCP4 suite [32].

b Numbers in parentheses refer to data in the highest resolution shell.

c *R_merge = _*Σ|I_h_−<I_h_>|/Σ I_h_, where I*_h_* is the observed intensity and <I*_h_*> is the average intensity.

d *R_work_* = Σ ||F_obs_|-k|F_cal_||/Σ|F_obs_|.

e *R_free_* is the same as *R_obs_* for a selected subset (10%) of the reflections that was not included in prior refinement calculations.

f Ordered residues: apo structure (Met1 to Tyr337 in subunit A and Ile3 to Ala317 in subunit B), a binary complex (Met1 to Tyr337 in subunit A and Ile3 to Lys315 in subunit B), and a ternary complex (Met1 to Asn338 in subunit A and Ser4 to Lys315 in subunit B).

g Outliers identified using a program MolProbity [33]: three residues in apo form, Ser333 for subunit A, Asn157, Asn192 for subunit B; two residues in the binary complex, Asn157 for subunit A, Asn157 for subunit B; four residues in the ternary complex, Arg113, Asn157 for subunit A, Met143, Asn157 for subunit B.

**Figure 2:**
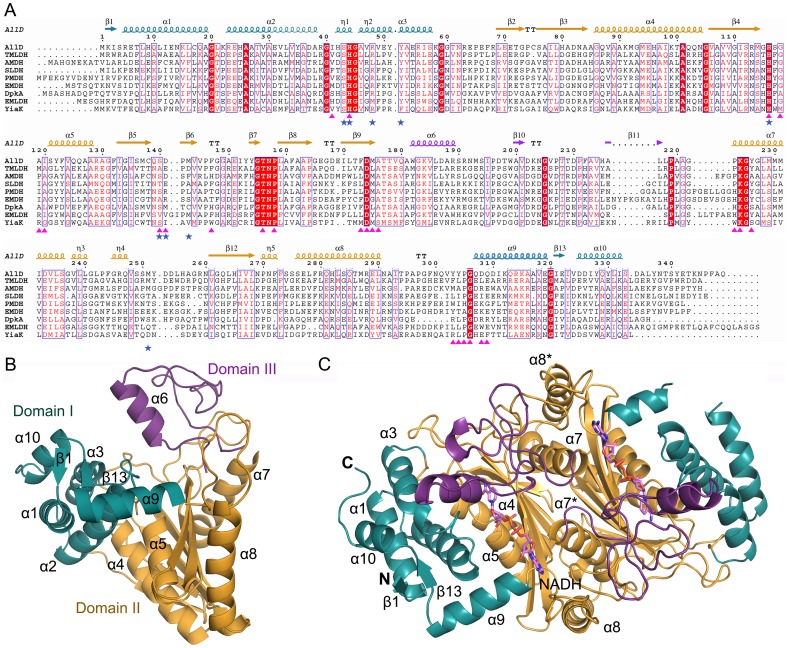
Sequence alignment and the overall conformation of AllD in the monomeric and dimeric structure. **A**, The amino acid sequences of AllD are compared with members of the NAD(P)H-dependent oxidoreductase family with known structures: TMLDH annotated as *Thermus thermophilus* HB8 Type 2 malate/lactate dehydrogenase (1VBI; Z-score, 45.3; rmsd, 1.5 Å), AMDH annotated as *Agrobacterium tumefaciens* malate dehydrogenase (1Z2I; Z-score, 44.3; rmsd, 1.5 Å), SLDH for *Methanocaldococcus* l-sulfolactate dehydrogenase (2X06; Z-score, 41.6; rmsd, 1.9 Å) [23], PMDH annotated as *Pyrococcus horikoshii* OT3 malate dehydrogenase (1V9N; Z-score, 41.1; rmsd, 1.6 Å), EMDH annotated as *Entamoeba histolytica* malate dehydrogenase (3I0P; Z-score, 40.6; rmsd, 2.1 Å), DpkA for *Pseudomonas syringae* Δ^1^-piperideine-2-carboxylate/Δ^1^-pyrroline-2-carboxylate reductase (2CWF; Z-score, 37.4; rmsd, 2.2 Å) [24], EMLDH annotated as *E. coli* malate/l-lactate dehydrogenases (2G8Y; Z-score, 37.0; rmsd, 2.4 Å), YiaK for *E. coli* 2,3-diketo-l-gulonate reductase (1S20; Z-score, 36.1; rmsd, 2.7 Å) [25]. Highly conserved residues are shown in red and boxed in blue; strictly conserved residues are shown on a red background. Red triangles represent the residues involved in binding of NADH at the active site, while residues for the glyoxylate-binding site are indicated by blue asterisks. The secondary structural elements defined in an apo form are shown for the corresponding AllD sequences, with Domains I, II, III in cyan, orange, and magenta, respectively. These color codes are used throughout the manuscript, and the figure was prepared using ESPript [31]. **B**, The overall structure of monomeric AllD is shown, displaying the secondary structure elements with each domain in different colors. The molecule was orientated so that the inter-domain interface is located at the center of the monomer. **C**, A dimer in the asymmetric unit of the binary complex with NADH is displayed, with helices in the intersubunit interface.

In the monomer, AllD has 10 α-helices and 13 β-strands, which fold into three distinct domains (Figure 2, A and B). Domain I contains N- and C-terminal regions and consists of a four-helix bundle (α1, α2, α3, and α10), two antiparallel short β-strands (β1 and β13), and helix α9. These elements are oriented so that the antiparallel β-strands, covered by helix α9, seal off one end of the four-helix bundle (Figure 2B). Domain II is the central folding unit, with seven β-strands (β2–β3–β4–β12–β5–β8–β9 in order) and four α-helices (α4, α5, α7, and α8). In this core structure, the antiparallel β-sheets are sandwiched between α-helices located on both sides of the β-sheet. Specifically, two parallel helices α4 and α5, which are located on the side of Domain I, are packed against the central β-sheet along the β-strands, while two other helices (α7 and α8) position on the opposite side of the β-sheet, with their helical axes perpendicular to that of the β-strands. In these interactions, α7, which faces Domain III, interacts with the residues at one end of the β-sheet, but α8 is detached from and placed at the other end of the central sheet, leaving the middle region of the sheet as a concaved, exposed surface (Figure 2B). Domain III is made up of loops, one helix (α6), and two short β-strands. Overall, Domains I and III, which protrude from Domain II and face each other, are localized on opposite sides of the central β-sheet in Domain II, and these domain orientations generate the inter-domain interface on the top of the central β-sheet surrounded by Domains I and III (Figure 2B).

In the dimer, Domain II from each subunit interacts extensively with each other, in a face-to-face orientation, between the central β-sheet and two helices α7 and α8 (Figure 2C). Moreover, these interactions are related by two-fold symmetry between the two monomers. In particular, dimerization is mediated by sliding the helix α7, which protrudes from one subunit, into the concaved, exposed surface of the central β-sheet in the other subunit, resulting in an interdigitate arrangement of α8, α7*, α7, and α8* from the two subunits (hereafter, an asterisk denotes an element or a residue from a different subunit). These helical arrangements generate a two-helix bundle in the intersubunit interface between α7 and α8, each from different subunits (Figure 2C), and these two helices are stabilized in the dimer mainly through hydrophobic interactions. In contrast, α7* and α7, which are embedded in the middle of the intersubunit interface, are distant from each other and do not interact. The dimerization interface is extensive and its buried surface area is calculated to be approximately 3040 Å^2^, which corresponds to 25% of the total monomer surface area.

### NADH Binding Site in the Binary Complex

**Figure 3:**
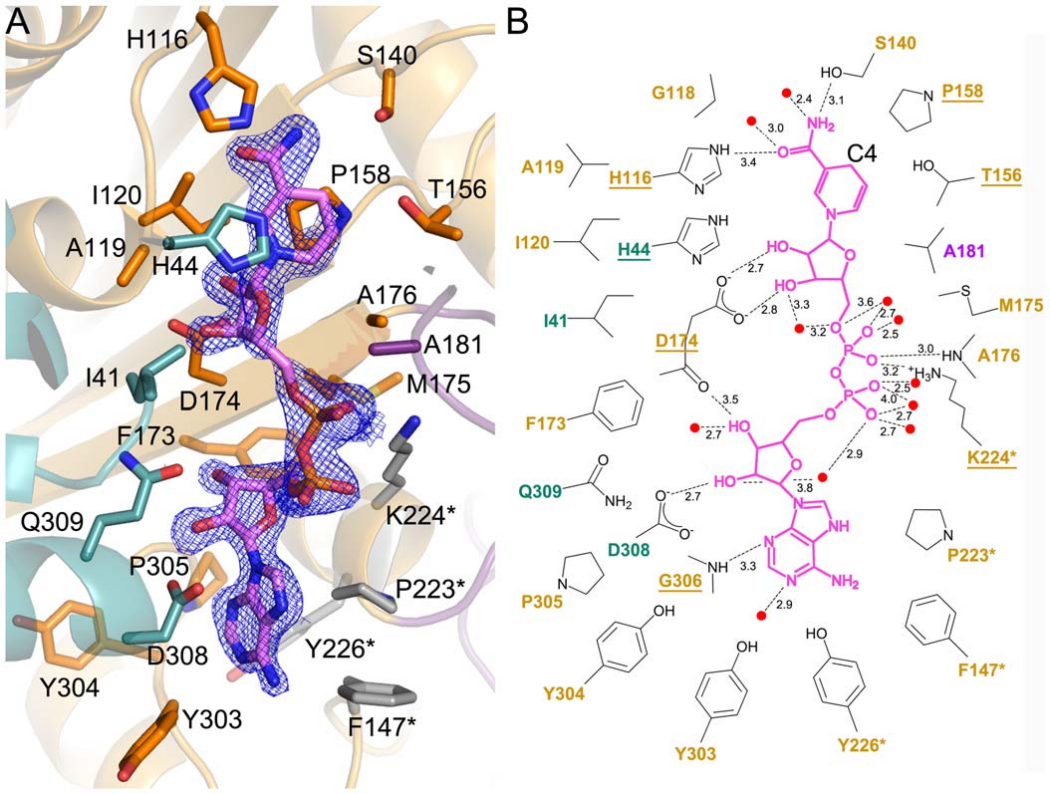
Interactions between NADH and AllD. **A**, NADH is located at the inter-domain interface, with the *Fo-Fc* electron density map contoured at 3σ. Residues for the NADH-binding site are indicated in Figure 2A and displayed by the color codes used in Figure 2B, except for residues in gray, which represent interactions between other subunits. **B**, Schematic diagram showing the NADH-binding mode in the active site. The dashed lines indicate putative hydrogen bonds, which are labeled with the interatomic distance in Å; other residues represent van der Waals interactions of less than 5.0 Å. Water molecules are shown as red spheres. Residues are indicated by color coding, and underlined if they are conserved within the family (see Figure 2A).

The structure for the binary complex of AllD with NADH (the AllD–NADH complex) revealed a binding pocket for a cofactor NADH which is localized mainly at the inter-domain interface of each subunit, and further stabilized by dimerization (Figures 2C and 3A). Specifically, NADH adopts an extended conformation and is located on top of the central β-sheet of Domain II, with its orientation perpendicular to that of the β-strands. The cofactor is surrounded by residues primarily from Domains I and II, and α7*. The nicotinamide ring faces toward the inner side of monomer, while the adenine moiety is located at the intersubunit interface. In total, 24 residues are involved in these interactions, including 15 residues from Domain II, four residues from Domain I, one residue from Domain III, and an additional four residues from Domain II* (Figure 3B). Among those interactions, 23 hydrogen bonds within a distance of 3.6 Å were identified, although nine are directly involved with the enzyme and the remaining are water-mediated. Other interacting residues mediate hydrophobic interactions at a distance of less than 5.0 Å.

NADH is buried in the pocket such that only 10% of the possible surface area is exposed. In particular, the nicotinamide ring, which acts as a hydride acceptor or donor during catalysis, is located on top of the N-terminus of β8 (Figure 3A), and its position appears to be stabilized by stacking interactions between Pro158 and His44, with further adjustments by hydrogen bonding between its carbonyl and amino group to His116 and Ser140, respectively. Notably, the *pro-*S face at C4 (*i.e*. B-face) of the nicotinamide ring is exposed to and accessible from the solvent, suggesting that this face functions as the putative substrate-binding site. In contrast, the adenine moiety at the opposite end of NADH does not exhibit notable stacking interactions. Its binding environment is characterized mainly by hydrophobic residues, including Tyr303, Tyr304, Pro305, Asp308, Phe147*, Pro223*, and Tyr226*. Additionally, the two ribose groups in NADH have hydrogen bonds between their hydroxyl groups and the nearby residues, such as Asp174 and Asp308, as well as water molecules (Figure 3B). More water-mediated hydrogen bonds are localized with the phosphate groups, possibly to neutralize the negative charges on phosphate, like Lys224* does.

### Binding of NADH and Glyoxylate in the Ternary Complex

An extensive soaking experiment designed to form a ternary complex of AllD with NADH and various ligands, including (*S*)-ureidoglycolate, oxalurate, and possible analogs such as hydantoic acid or malic acid, was not fully successful under various conditions. However, we were able to identify the binding site of glyoxylate in the vicinity of NADH when a crystal was soaked with oxalurate. In particular, a NADH cofactor is located at the site identical to that in the binary complex. The formation of glyoxylate could have occurred through the spontaneous degradation of oxalurate under our experimental conditions, and its binding site could provide the binding mode of (*S*)-ureidoglycolate substrate.

**Figure 4:**
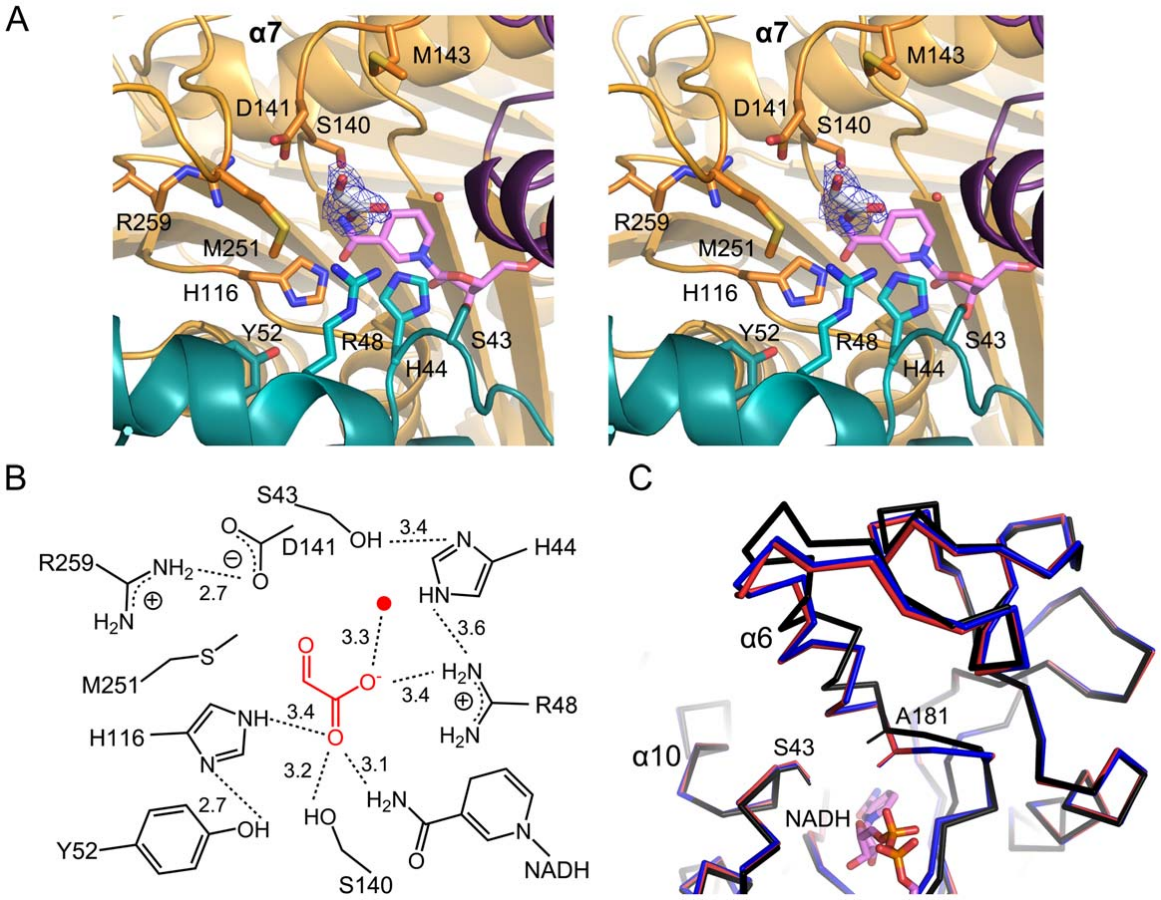
Binding site for glyoxylate and NADH-induced conformational changes. **A**, Glyoxylate, with a *Fo-Fc* electron density map contoured at 1σ, is shown with the nearby residues within a distance of 5.0 Å. NADH is indicated in magenta. Ser43 and Tyr52 form hydrogen bonds with His44 and His116, respectively. **B**, Schematic view for the binding site of glyoxylate. In this scheme, glyoxylate-interacting residues in the first and second shell are shown, along with the possible hydrogen-bonding network in those residues. Water molecules are indicated by the red circle, as seen in a. **C**, Conformational changes are observed in Domain III of the binary (red) and ternary (blue) complex compared to that in the apo form (black).

The electron density map for glyoxylate was unambiguous in subunit A, but not in subunit B (Figure 4A). We could distinguish the position of the aldehyde carbonyl group from the carboxyl group of glyoxylate (Figure 4A). The planar molecule glyoxylate is 4.0–5.0 Å over the B-face of the nicotinamide, but with a perpendicular orientation to it. Specifically, the carboxylate group interacts with Arg48 within a distance of 3.4 Å, and its position is further stabilized by hydrogen bonds at a distance of 3.1–3.4 Å to His116, Ser140, and the amino group of the nicotinamide ring (Figure 4B). In contrast, the aldehyde carbonyl oxygen orients toward the opposite direction of the nicotimamide ring, and is within 3.9 Å of the nearby residues Asp141 and Met251. Not only these residues, which create a glyoxylate-interacting first shell, but Ser43, His44, Tyr52, and Arg259 also interact with these first-shell residues through a hydrogen-bonding network.

### Structural Comparison

To characterize possible conformational changes induced by the binding of NADH or/and glyoxylate, we compared the three-dimensional structures by superposition. First, conformational differences were investigated between two subunits of AllD in the apo form. This analysis resulted in a root-mean-square deviation (rmsd) of 0.45 Å for 315 corresponding Cα atoms, and these differences were evenly distributed throughout the structure, indicating that the structure of subunit A and B are essentially identical. Further analysis was focused on subunit A of the binary and ternary complexes because this particular subunit contained glyoxylate in the ternary complex.

The apo structure differed from both the binary and ternary complex by rmsd values of 0.54 Å for 337 Cα atoms. The two complexes exhibited essentially identical conformations, within rmsd values of 0.19 Å for 337 Cα atoms. Although the rmsd values between the apo form and the two complexes were not significant, large differences are localized only in Domain III. Specifically, in the binary complex, binding of NADH into the active site caused a rigid-body movement of Domain III toward Domain II at the inter-domain interface (Figure 4C); residues Val179 to Ser194 containing a helix α6 in Domain III showed large displacements of Cα atoms in the range of 0.9–3.0 Å. Along with these changes in Cα atoms, positional shifts of the side chains in this region altered the interactions with NADH, as well as with Domain II. Compared to the apo structure, the side chain of His44 moved out by 1.2 Å, accommodating NADH and forming a possible stacking interaction with the nicotinamide ring, while Asp174 rotated its side chain as much as 4.0 Å toward NADH and hydrogen bonded with the hydroxyl group of ribose, adjacent to the nicotinamide ring (Figure 3, A and B). Additionally, the carboxylate group of Asp308 moved approximately 1.0 Å, forming a hydrogen bond with the hydroxyl group of ribose adjacent to the adenine base. In the ternary complex, further changes were not observed, except for Asp141, whose side chain rotated as much as 3.9 Å toward glyoxylate.

Overall, AllD undergoes modest conformational changes in response to cofactor binding by moving Domain III toward the active site, which could be characterized by the induced-fit changes. Those movements cause the side chains of residues in the active site to take a productive orientation, allowing interactions with the incoming NADH or glyoxylate.

### Functional Analysis

Although the ternary complex with NADH and glyoxylate indicates the binding site of the ligand, it is unlikely that the catalytic binding environment of glyoxylate fully reflect that of (*S*)-ureidoglycolate. In particular, structural analyses suggest that the glyoxylate aldehyde carbon atom corresponds to the chiral, hydroxyl-bearing carbon atom of (*S*)-ureidoglycolate (Figure 1). However, the aldehyde carbon of glyoxylate is too distant from the C4 atom of NADH (about 6.0 Å) for the proposed hydride transfer reaction (Figure 4A). The glyoxylate binding mode also suggests that Asp141 could act as a general base, attracting a proton from the hydroxyl group at the chiral carbon of (*S*)-ureidoglycolate. Therefore, kinetic analyses were performed using various mutant enzymes to characterize the functional roles of those residues proximal to the binding site of glyoxylate.

We selected nine residues for site-directed mutagenesis (Figure 4B): five that directly interact (within 4.5 Å) with glyoxylate including Arg48, His116, Ser140, Asp141, and Met251, and four that possibly form a hydrogen-bonding network with the glyoxylate-interacting, first shell residues including Ser43, His44, Tyr52, and Arg259. Purified enzymes were assumed to be in a native conformation based on circular dichroism spectra similar to those of the wild-type AllD (Figure S3). Kinetic parameters for various AllD enzymes using (*S*)-ureidoglycolate and NAD^+^ as substrates are listed in Table 2 and Figure S4. Among the first shell residues, it is notable that the R48A and H116A mutants were completely inactive. Other mutants maintained their activity with 5- to 13-fold increases in *K*_m_ and 1.2- to 44-fold reductions in *k_cat_* relative to the wild-type enzymes, which is consistent with the structural analysis of glyoxylate binding site (Figure 4, A and B). In particular, three different mutants of a putative general base Asp141 failed to eliminate AllD activity and instead resulted in 5- to 13-fold increases in *K*_m_. These observations suggest that the first shell residues play a pivotal role in the binding of (*S*)-ureidoglycolate to the AllD active site and/or catalysis. Under these conditions, Asp141 does not act as a general base, but is instead involved in the binding of substrate. Therefore, Arg48 and His116 are essential for substrate binding and/or catalysis (see Discussion).

**Table 2:** Enzyme assay of the wild-type AllD and its mutants.

		*K*_m_ (mM)	*k*_cat_ (s^−1^)	*k*_cat_/*K*_m_ (mM^−1^s^−1^)
Wild-type	(*S*)-ureidoglycolate	1.06 (0.08)^a^	57.06 (1.54)	54.3
	NAD^+^	0.56 (0.04)	62.39 (1.14)	110
S43A	(*S*)-ureidoglycolate	10.75 (1.01)	1.37 (0.06)	0.13
	NAD^+^	2.28 (2.35)	2.51 (0.23)	1.10
H44A	(*S*)-ureidoglycolate	16.94 (2.04)	1.76 (0.12)	0.10
	NAD^+^	0.65 (0.11)	1.03 (0.05)	1.6
R48A	(*S*)-ureidoglycolate	ND b	−	−
	NAD^+^	−	−	−
Y52F	(*S*)-ureidoglycolate	16.82 (3.92)	1.92 (0.28)	0.11
	NAD^+^	1.26 (2.19)	1.90 (0.14)	1.51
H116A	(*S*)-ureidoglycolate	ND b	−	−
	NAD^+^	−	−	−
S140A	(*S*)-ureidoglycolate	12.38 (2.19)	1.29 (0.12)	0.10
	NAD^+^	1.39 (0.20)	0.02 (0.00)	0.01
D141A	(*S*)-ureidoglycolate	5.27 (0.46)	1.62 (0.05)	0.31
	NAD^+^	0.52 (0.04)	1.06 (0.02)	2.04
D141N	(*S*)-ureidoglycolate	5.84 (0.25)	1.97 (0.03)	0.34
	NAD^+^	0.81 (0.07)	1.47 (0.03)	1.81
D141E	(*S*)-ureidoglycolate	14.13 (2.37)	1.67 (0.15)	0.12
	NAD^+^	0.37 (0.03)	0.87 (0.02)	2.35
M251A	(*S*)-ureidoglycolate	13.15 (1.62)	45.98 (3.32)	3.49
	NAD^+^	0.93 (0.06)	30.65 (0.55)	32.9
R259A	(*S*)-ureidoglycolate	11.47 (1.25)	11.98 (0.66)	1.04
	NAD^+^	1.49 (0.07)	9.29 (0.13)	6.23

a Values in parentheses are standard error.

b Activity was not detected even at 1.5 µM (50 µg/mL) of enzyme.

Further kinetic analyses showed similar catalytic features for second shell residues of the mutant enzymes, with large (10- to 16-fold) increases in *K*_m_ and significant (29- to 42-fold) reductions in *k_cat_*, with exception of the R259A mutant. In the R259A mutant enzyme, the *k_cat_* value was approximately 21% that of the wild-type enzyme, with about an 11-fold increase in *K*_m_. Together with structural analyses, these results suggest that the second shell residues also contribute to activity, possibly by stabilizing the side chain orientations of the first shell residues for the productive binding mode of the incoming (*S*)-ureidoglycolate and subsequent catalysis. Additional kinetic measurements against NAD^+^ indicated that all mutations did not significantly affect the *K*_m_ values of NAD^+^ to AllD, which rules out the possibility that the reduced activity observed for the mutant enzymes is due to changes in the binding affinity of NAD^+^ to the enzyme.

## Discussion

(*S*)-Ureidoglycolate dehydrogenase AllD belongs to one of eight clades of l-sulfolactate dehydrogenase-like superfamily [18], [20]. Members of this superfamily were initially annotated as type 2 malate/l-lactate dehydrogenases [19], but differed from the conventional Rossman-fold NAD(P)H-dependent malate/l-lactate dehydrogenases [21] in their amino acid sequences, substrate specificities, and structural features. Structural homology searches using the program DALI [22] with subunit A of AllD in its apo form as a search model indicated nine structures in this family (Figure 2A), with Z scores of 36–54 and rmsd values of 0.7–2.7 Å for Cα atoms. Among these enzymes, the structures of six proteins were determined by the Structural Genomics Initiative in the absence of their biochemical roles (see Figure legend to Figure 2A). In particular, a search model (PDB code 1XRH) for a molecular replacement in this study was essentially identical with AllD, with a Z score of 53.9 and rmsd of 0.7 Å. Three enzymes were characterized for their substrates: l-sulfolactate dehydrogenase (PDB code 2X06) [23], Δ^1^-piperideine-2-carboxylate/Δ^1^-pyrroline-2-carboxylate reductase (DpkA; PDB code 2CWF) [24], and 2,3-diketo-l-gulonate reductase (YiaK; PDB code 1S20) [25]. Structural comparison of AllD with those nine structures revealed that members of this family share common features for dimerization and an overall structure with three domains. In addition, the NADH cofactor adopts an extended conformation [23]–[25], which is clearly distinguishable from a bent conformation usually associated with other NAD(P)H-dependent oxidoreductases. Furthermore, structural analyses of DpkA and YiaK also indicated ligand-induced conformational changes and the binding location for NAD(P)H with the exposed B-face of the nicotinamide ring [24], [25]. In particular, conserved residues exist in the cofactor-binding site: His44, His116, Thr156, Pro158, Asp174, Lys224, and Gly306 (Figure 3B). These residues are involved in stabilizing the nicotinamide ring, ribose, and the negatively charged phosphate groups, either by hydrophobic interactions or by hydrogen bonding, serving as the main structural elements during cofactor binding.

Comparison of the AllD ternary complex with that of DpkA in complex with NADPH and a substrate analog (PDB code 2CWH) [24] provides a structural basis for the preference of NAD^+^ over NADP^+^ as an AllD cofactor (Figure S5). In DpkA, ribose 2′-phosphate groups of NADPH bound to a cluster of arginine residues (Arg314 and Arg315) in the α9 of Domain I. Structurally equivalent residues in AllD for the arginine cluster are Asp308 and Gln309 (Figure 2A). In addition to these sequence changes, differences in the orientation of α9 in Domain I allows those two AllD residues to preoccupy the putative ribose 2′-phosphate group-binding site in NADPH. As a result, Asp308 in AllD mediates hydrogen bonding to the 2′-hydroxyl group of ribose in NADH (Figure 3B). Therefore, charge repulsion by sequence variation, which was predicted by Goto *et al.* [24], and possible steric hindrance due to conformational differences in this region, are likely major elements to abolish binding of NADP^+^ to AllD.

Structures of YiaK [25] and DpkA [24] were characterized in the presence of an inhibitor and a substrate analog, respectively. A common feature of these two ligands is the location of the ligand-binding sites, although details of their interactions are different for each enzyme (Figure S6). Generally, the ligand binding sites are positioned over the B-face of the nicotinamide ring, and the ligand reaction site projects onto the C4 atom of the ring. Due to these stereochemical restraints, *pro*-S hydrogen transfer to and from C4 of the nicotinamide ring was proposed as a catalysis mechanism for these two enzymes. Furthermore, a histidine residue corresponding to either His44 or His116 in AllD, which are both located in close proximity to the nicotinamide ring of NADH (Figure 4A), was proposed as the catalytic residue. In DpkA, a general acid catalyst was assigned to a histidine equivalent to His44 in AllD [24], while either His44 or His116 in AllD was proposed as a general catalytic base in YiaK [25]. These assignments are, however, based on interactions of the ligand with its surrounding environment and proximal residues in the absence of further biochemical evidence.

Based on the structural and functional analyses described herein, we propose a mechanism of AllD-dependent oxidation of (*S*)-ureidoglycolate into oxalurate. Analogous to that of YiaK and DpkA, an enzyme-mediated deprotonation step most likely occurs by abstracting a proton from the hydroxyl group attached to the chiral carbon, followed by electron migration. Concurrently, a hydride transfer is carried out from the chiral carbon to the C4 atom on the B-face of the nicotinamide ring. Functional analyses (Table 2) indicate that His116, instead of Asp141, appears to act as a general catalytic base and that Arg48 plays a crucial role in stabilizing the binding of (*S*)-ureidoglycolate. Since these functional features differ from those in the ternary AllD–NADH–glyoxylate complex (Figure 4B), we modeled the binding mode of (*S*)-ureidoglycolate, on the basis of the functional analysis and proposed mechanism (Figure S7).

The coordinates for (*S*)-ureidoglycolate used in this modeling study are based on those of (*S*)-ureidoglycine [14], but modified to have the hydroxyl group for (*S*)-configuration (Figure S1). We manually placed the hydrogen of a chiral carbon atom onto the C4 atom of the nicotinamide ring, and oriented the hydroxyl group at a chiral carbon near His116, dictating the relative orientation of the carboxylate group and the ureido tail. Specifically, an energy-minimization step in the program CNS [26] indicated that the placement of (*S*)-ureidoglycolate did not cause any noticeable structural rearrangement of the side chain for active site residues. The carboxylate group is positioned within hydrogen-bonding distance (less than 3.0 Å) of Ser140 and Asp141, and at an appropriate distance for Van der Waals interactions (about 4.5 Å) from Met251. The ureido tail is proximal to Arg48 and is available for hydrogen bonding within a distance of 3.4 Å. In addition, the chiral carbon is located 4.0 Å from the C4 atom of the nicotinamide ring and the side chain of His116 is within 3.0 Å of the hydroxyl group at a chiral carbon. This modeling is consistent with the functional analyses performed in this study and agrees with residue conservation. Arg48, Ser140, and Asp141 are highly conserved among AllD-like enzymes from various prokaryotic organisms (Figure S8) but not in the novel NAD(P)H-dependent oxidoreductase family, which displays different substrate specificities (Figure 2A). The validity of the binding mode of (*S*)-ureidoglycolate is further supported by a kinetic analysis of Asp141. The D141E mutant enzyme shows a two- to three-fold increase in *K*_m_ relative to that of the D141A and D141N mutant enzymes (Table 2). This may be due to unfavorable interactions with the carboxylate group of (*S*)-ureidoglycolate caused by a larger side chain in the D141E mutant. When the binding mode of a modeled (*S*)-ureidoglycolate is compared with that of glyoxylate, both ligands occupy almost identical sites but the relative orientation of the carboxylate group is completely different (Figure S7). The carboxylate group of glyoxylate points toward Arg48 instead of Ser140 and Asp141 (Figure 4A). Therefore, the binding mode of glyoxylate is apparently different from that of the substrate, although the glyoxylate moiety provides general information about the substrate binding site. Other residues in the active site remain in environments identical to those in the ternary AllD–NADH– glyoxylate complex. Therefore, our functional assignments on other active site residues are consistent with the structural analyses of this study. In particular, the second shell residues play an essential role in catalysis by maintaining the side chains of the first shell residues of the active site in catalytically active orientations.

Our assignment of His116 as a general catalytic base is supported by conservation of this particular histidine residue in AllD-like enzymes from various organisms (Figure S8). This identification, along with the proposed catalytic residue in YiaK and DpkA, also suggests that all members of the l-sulfolactate dehydrogenase-like superfamily utilize a histidine residue, corresponding to either the His116 or His44 in AllD, near the nicotinamide ring of the cofactor as a catalytic residue. Indeed, these two histidine residues are invariant in this family (Figure 2A). Involvement of a particular histidine residue in catalysis likely depends on the binding mode of the substrate, which could differ depending on the chemical nature of the active site in each enzyme. We have been unable to crystallize AllD mutants at position 116, including H116A, H116N, and H116Q, for soaking experiments with the (*S*)-ureidoglycolate substrate or oxalurate product.

In this study, we determined the crystal structures of *E. coli* (*S*)-ureidoglycolate dehydrogenase AllD, which forms oxalurate in the presence of NAD^+^. This structure represents a metabolic branch point enzyme during (*S*)-ureidoglycolate utilization, which is the end product of purine catabolism common to plants, as well as some bacteria and fungi. Structure determination in the apo form, and the binary and ternary complex reveals a novel fold for the NADH-binding domain, a conformational change, and the binding of glyoxylate. Further kinetic analysis provided the functional roles of the active site residues, as well as a possible catalytic mechanism.

## Materials and Methods

### Cloning, Expression, and Purification

The *allD* gene from *E. coli str. K-12 substr. DH10b* (GenBank Accession Number NC_010473) was amplified using PCR with sequence-specific primers (Table S1). The amplified DNA product was ligated into the expression vector pET28b (Merck), which was modified to contain a tobacco etch virus cleavage site between a His-tag and the multicloning sites, and its sequences were verified.

Recombinant AllD with the N-terminal His-tag was expressed in *E. coli* BL21(DE3) (Merck). Cells harboring the AllD plasmid were grown at 37°C in Luria–Bertani medium containing 10 mg/L kanamycin to an OD_600_ of 0.8, and then induced at 22°C for 12 h by adding 1 mM isopropyl-β-d-thiogalactopyranoside. The harvested cells were resuspended and sonicated in buffer A [50 mM NaH_2_PO_4_ (pH 7.5) and 500 mM NaCl]. The N-terminal His-tagged AllD was purified from the cell lysate using immobilized metal affinity chromatography with buffer A plus 500 mM imidazole. After dialysis against buffer B [50 mM Tris–HCl (pH 7.5) and 1 mM DTT], the His-tag was removed by treatment with tobacco etch virus protease in buffer B plus 5 mM DTT, followed by additional immobilized metal affinity chromatography with buffer B. The purified AllD was concentrated to approximately 14 mg/mL for crystallization, with its molar extinction coefficient of 31,860 M^−1 ^cm^−1^ at 280 nm.

For functional analysis, various AllD mutants were produced by site-directed mutagenesis using a QuikChange Kit (Agilent), with mutagenic primers (Table S1). Their expression and purifications were identical to the procedures described above, except that the N-terminal His-tag was not removed, and the resulting mutant enzymes were concentrated to 4 mg/mL for functional analysis. Allantoinase [12], allantoate amidohydrolase (AAH), and (*S*)-ureidoglycine aminohydrolase (UGlyAH) (Figure S1) were also expressed and purified to catalyze the conversion of allantoin to (*S*)-ureidoglycolate as reported previously [14].

### Crystallization

Initially, AllD in the absence of the cofactor was crystallized at 22°C using the hanging-drop vapor-diffusion method in a crystallization buffer consisting of 0.1 M MES (pH 6.0) and 4.0 M NaCl. Crystals of the binary AllD–NADH complex and the ternary AllD–NADH– glyoxylate complex were obtained through a crystal-soaking experiment. In particular, the binary complex was obtained by soaking AllD crystals for 10 min in a solution of 100 mM NADH in 50 mM Tris–HCl (pH 8.1) and crystallization buffer. For the ternary complex, a crystal of AllD was presoaked in 100 mM NADH for 10 min, and subsequently soaked for an additional 20 min in a solution containing 100 mM NADH in 50 mM Tris–HCl (pH 8.1), crystallization buffer, and ligand (see below). Soaking the crystal for less than 20 min did not allow for ligand binding.

Substrate (*S*)-ureidoglycolate was produced in the presence of AAH and UGlyAH (Figures S1 and S9). The reaction mixture contained 7.5 mM allantoic acid (Sigma-Aldrich), AAH (2.07 µM), and UGlyAH (0.69 µM) and was kept at 30°C in 100 mM Tris–HCl (pH 8.1) and 100 µM MnCl_2_ [13], [14]. In order to produce the oxalurate product, AllD (9.94 µM) and 3 mM NAD^+^ were added to the reaction mixture after the formation of (*S*)-ureidoglycolate was verified (Figure S9). Later, we found that the binding of (*S*)-ureidoglycolate was not characterized under these experimental conditions. However, one molecule of glyoxylate was observed in the vicinity of the active site after crystal was soaked in an oxalurate solution.

### Data Collection and Structure Determination

Data collection at 100 K was carried out at the Pohang Accelerator Laboratory, Pohang, Korea, on beamlines 4A, 5A, and 6C. Single-wavelength data for the apo form, the AllD–NADH binary complex, and the AllD–NADH–glyoxylate ternary complex were collected at 2.13 Å, 1.64 Å, and 1.77 Å, respectively (Table 1). The crystals were cryoprotected by adding 25% glycerol to each crystallization solution. The program HKL2000 [27] was used for data processing and all crystals belong to space group P*4_2_2_1_2*, with two monomers per asymmetric unit (Table 1).

The structure of the apo form of AllD was solved by molecular replacement using the program PHENIX [28] with a monomer of *E. coli* AllD (PDB code 1XRH) as the search model. Manual model building and refinement were performed using the programs COOT [29] and PHENIX, respectively. After several iterative cycles of manual inspection and refinement, the model was built including Met1 to Tyr337 in subunit A and Ile3 to Ala317 in subunit B; the density for the C-terminal twenty residues in subunit B was too disordered to allow model building (Table 1). This model was then used as an initial structure to determine the AllD–NADH binary complex. Specifically, the program PHENIX was used to perform three macro-cycles of a refinement, each with bulk-solvent and anisotropic scaling, individual coordinates and isotropic B-factors refinement, and refinement of occupancies. The resulting electron density map clearly showed one molecule of NADH bound in each subunit. Subsequently, the AllD–NADH–glyoxylate ternary complex was determined using a refined structure of the binary complex as a starting model. In subunit A of the ternary complex, the electron density map corresponding to one molecule of glyoxylate was clearly identified in the vicinity of the NADH-binding region, while at the corresponding region of subunit B, the density for glyoxylate was highly disordered and its model was not included in the final structure.

In the final refinement stages for all three structures, TLS refinement was carried out using multiple TLS groups identified automatically in PHENIX. During TLS refinement, water molecules, whose refined temperature factors are less than 50 Å^2^, were assigned based on the possible hydrogen bonds to the enzyme or a nearby water molecule. Details of the refinement are described in Table 1. Unlike the apo structure, the electron density associated with the C-terminal residues (Ala316 to Tyr337) in subunit B appeared but ambiguous and disordered, and those residues were not included in subunit B for the binary and ternary complexes. Therefore, *R*_work_ and *R*_free_ values for the complexes were relatively high, even with the higher resolution data (Table 1).

Structure comparison and analyses were carried out using the program Superimpose in CCP4 suite [30], and the figures were prepared using PyMOL (DeLano, W.L., The PyMOL Molecular Graphics System).

### Functional Analysis

Enzyme assays were performed at 30°C using a UV-visible spectrophotometer (Jasco model V-560) equipped with a cuvette holder connected to a temperature-controlling water circulator. The reaction mixture of 2 mL contained (*S*)-ureidoglycolate and NAD^+^. After 20 s of incubation, the reaction was initiated by adding AllD. (*S*)-Ureidoglycolate was prepared as described previously [14]. To measure the steady-state kinetic parameters for (*S*)-ureidoglycolate as a substrate, a saturating concentration of NAD^+^ (3 mM, *i.e.*, about five-fold of the *K*_m_ value) was used, whereas 15.3 mM (*S*)-ureidoglycolate was added to measure the kinetic parameters of NAD^+^. The initial rate of NADH formation was determined by measuring the increase in absorbance at 340 nm for the first 20–30 s, assuming a molar extinction coefficient of 6,220 M^−1 ^cm^−1^ at 340 nm. *K*_m_ and *V*_max_ values were obtained using SigmaPlot, and *k*_cat_ values were computed by dividing *V*_max_ by the enzyme concentration used in the assay.

### Footnotes

Data deposition footnote: The atomic coordinates and structure factors (PDB code 4FJS for the apo form, 4H8A for the AllD-NADH binary complex, and 4FJU for the AllD-NADH-glyoxylate ternary complex) have been deposited in the Protein Data Bank (http://www.rcsb.org/).

## Supporting Information

Figure S1 Scheme for the conversion of (*S*)-allantoin to (*S*)-ureidoglycolate.
(PDF)

Figure S2 Kinetic analysis of AllD using NAD^+^ and NADP^+^. In this assay, 0.3 mM NAD^+^ or NADP^+^, AAH (2.07 µM;105.2 µg/mL), and UGlyAH (0.69 µM; 22.3 µg/mL) were incubated, followed by the addition of 0.15 mM allantoate to produce (*S*)-ureidoglycolate (see Figure S1). After 3 min, AllD (20 µg/mL) was added to initiate the reaction. In this figure, a reaction was recorded after a 3-min incubation, such that the peak in absorbance corresponds to the addition of AllD to a reaction mixture.
(PDF)

Figure S3 Circular dichroism spectra of the wild-type and mutant AllD enzymes. Circular dichroism was measured in a 10-mm path-length cuvette with a Jasco J-810 spectropolarimeter, using an enzyme concentration of 2 mg/mL in 20 mM Tris–HCl (pH 7.6) and 150 mM NaCl.
(PDF)

Figure S4 *K*_m_ and *V*_max_ values, and concentrations of each mutant used in this study. Fitting of the initial rate was carried out using the program SigmaPlot. Figures for the initial rate as a function of NAD concentration were essentially identical among all mutant enzymes, such that only one fitting is shown using the wild-type enzyme.
(PDF)

Figure S5 Stereoview of the binding site of NADH and NADPH. The ternary complex of AllD (yellow) and DpkA in complex with NADPH and a substrate analog (PDB code 2CWH) [24] (blue) were superimposed.
(PDF)

Figure S6 Binding site for ligand in AllD, YiaK, and DpkA. Ligand-binding sites are shown for the ternary complex of AllD with glyoxylate (yellow), YiaK with an inhibitor (magenta; PDB code 1S20) [25], and DpkA with a substrate analog (blue; PDB code 2CWH) [24]. It is noticeable that histidine residue corresponding to His116 in AllD is present in a structure of DpkA, but its orientation is quite different from that in AllD and YiaK.
(PDF)

Figure S7 The proposed binding mode of (S)-ureidoglycolate. We modeled the binding mode of (*S*)-ureidoglycolate (gray), based on the functional analysis and a proposed mechanism. The coordinates are based on those of (*S*)-ureidoglycine [14], and after placed in the active site, the model was subject to an energy-minimization step in the program CNS [26]. For comparison, the model for glyoxylate (green) and NADH (magenta) in the ternary complex is indicated. Details for the interactions are described in the text.
(PDF)

Figure S8 Sequence alignment of (*S*)-ureidoglycolate dehydrogenases annotated from various microorganisms. Gene access number is given in parentheses and several active site residues are indicated.
(PDF)

Figure S9 Formation of (*S*)-ureidoglycolate. This measurement was performed according to the procedures described previously [13], [14]. Accordingly, 2.5 mM α-ketoglutarate, 0.3 mM NADPH, 5 units of glutamate degydrogenase (Sigma-Aldrich), and AAH (2.07 µM; 105.2 µg/mL) were incubated, then 0.15 mM allantoate (Sigma-Aldrich) and UGlyAH (0.69 µM; 22.3 µg/mL) were subsequently added to the reaction mixture (shown in black). Absorbance decrease at 340 nm by addition of allantoate and UGlyAH is due to the released ammonia in each reaction, representing conversion of NADPH to NADP^+^. In an alternate experiment (red), UGlyAH was included in the pre-reaction mixture, followed by initiating the reaction with 0.15 mM allantoate. After approximately 3 min, the reaction was completed. Our calculation for the conversion of NADPH into NADP^+^, with a molar extinction coefficient of 6220 M^−1 ^cm^−1^ at 340 nm for NADPH, indicated a complete conversion of allantoate into (*S*)-ureidoglycolate.
(PDF)

Table S1 Primer sequences used in this study.
(PDF)
