# Supplementary figures and images for: Structural and Functional Insights into (S)-Ureidoglycolate Dehydrogenase, a Metabolic Branch Point Enzyme in Nitrogen Utilization
Source: PLoS One. 2012 Dec 20;7(12):e52066. doi: 10.1371/journal.pone.0052066 (PMC3527362; doi:10.1371/journal.pone.0052066)

Figure S1

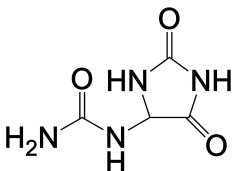

S-(+)-Allantoin

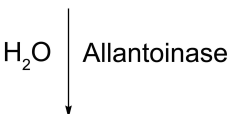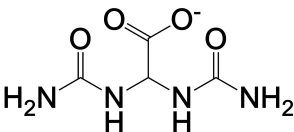

Allantoate

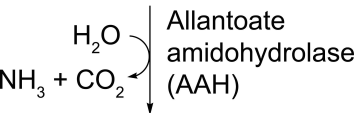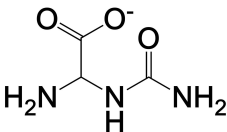

S-(-)-Ureidoglycine

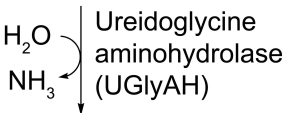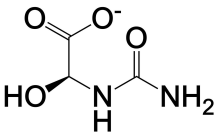

S-(-)-Ureidoglycolate

Supplement: Figure S1 — Scheme for the conversion of (S)-allantoin to (S)-ureidoglycolate. (PDF) [file pone.0052066.s001.pdf]

Figure S2

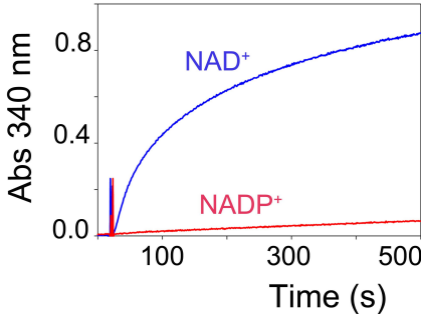

Supplement: Figure S2 — Kinetic analysis of AllD using NAD+ and NADP+. In this assay, 0.3 mM NAD+ or NADP+, AAH (2.07 µM;105.2 µg/mL), and UGlyAH (0.69 µM; 22.3 µg/mL) were incubated, followed by the addition of 0.15 mM allantoate to produce (S)-ureidoglycolate (see Figure S1). After 3 min, AllD (20 µg/mL) was added to initiate the reaction. In this figure, a reaction was recorded after a 3-min incubation, such that the peak in absorbance corresponds to the addition of AllD to a reaction mixture. (PDF) [file pone.0052066.s002.pdf]

Figure S3

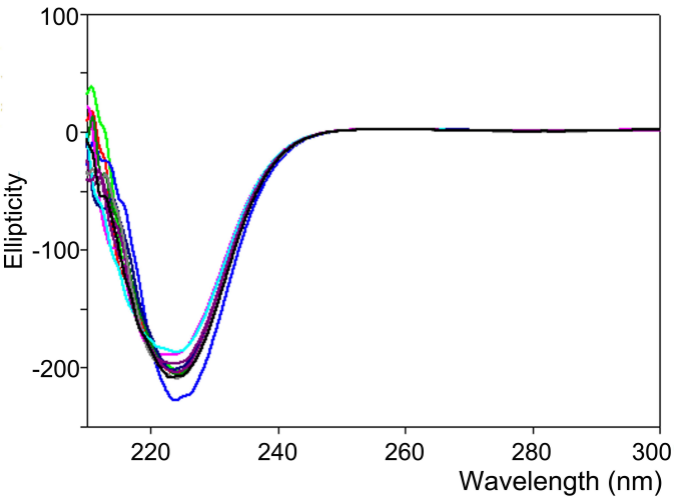

Supplement: Figure S3 — Circular dichroism spectra of the wild-type and mutant AllD enzymes. Circular dichroism was measured in a 10-mm path-length cuvette with a Jasco J-810 spectropolarimeter, using an enzyme concentration of 2 mg/mL in 20 mM Tris–HCl (pH 7.6) and 150 mM NaCl. (PDF) [file pone.0052066.s003.pdf]

Figure S4

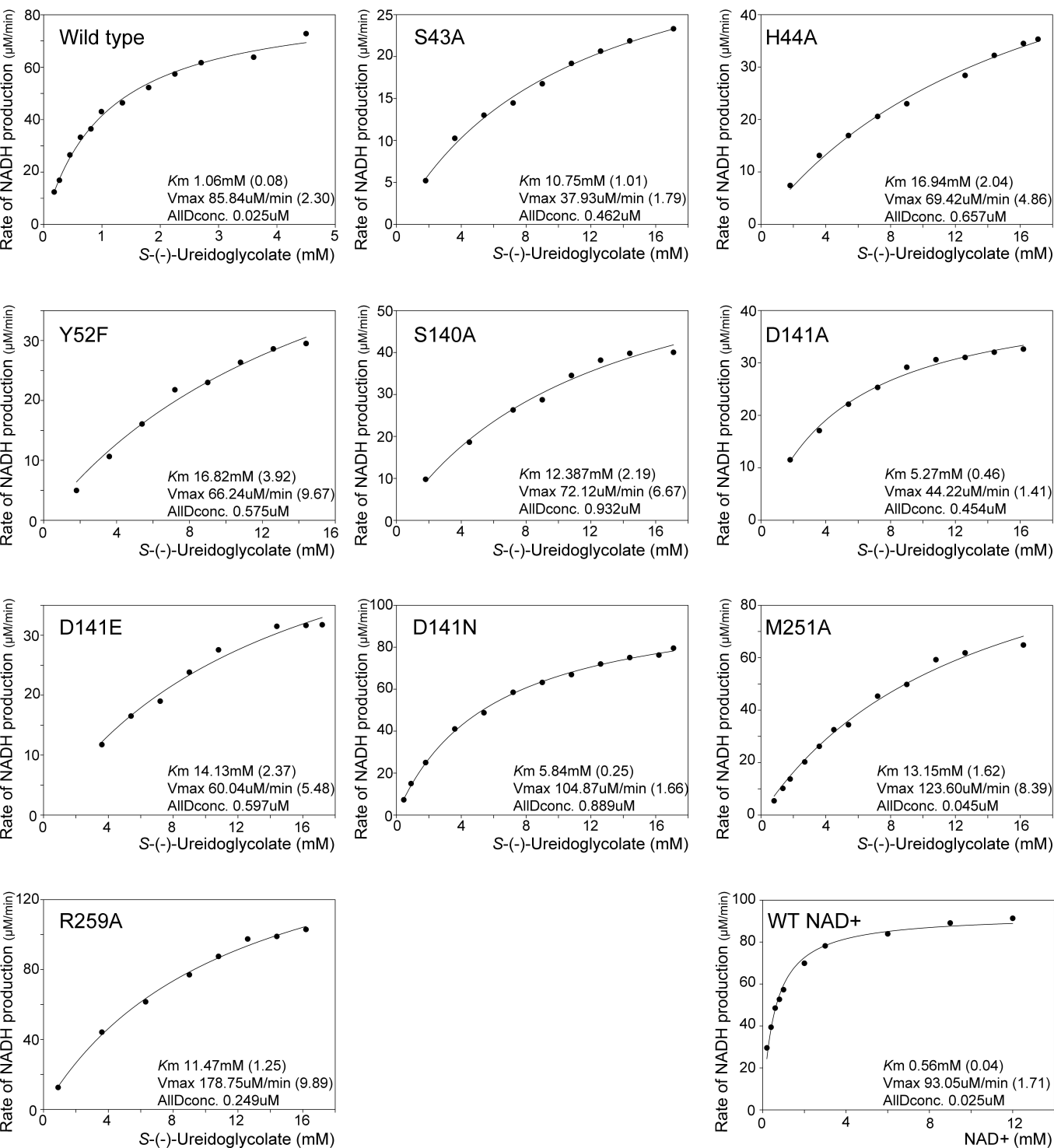

Supplement: Figure S4 — K m and V max values, and concentrations of each mutant used in this study. Fitting of the initial rate was carried out using the program SigmaPlot. Figures for the initial rate as a function of NAD concentration were essentially identical among all mutant enzymes, such that only one fitting is shown using the wild-type enzyme. (PDF) [file pone.0052066.s004.pdf]

Figure S5

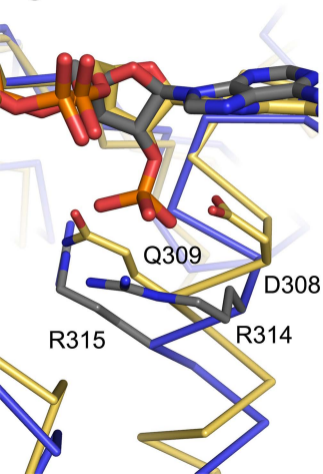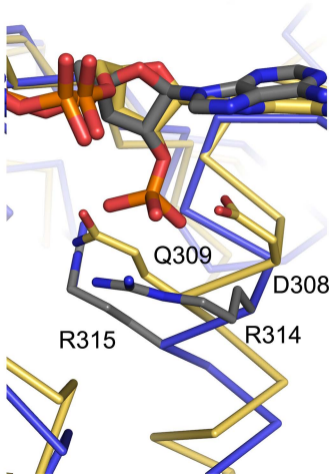

Supplement: Figure S5 — Stereoview of the binding site of NADH and NADPH. The ternary complex of AllD (yellow) and DpkA in complex with NADPH and a substrate analog (PDB code 2CWH) [24] (blue) were superimposed. (PDF) [file pone.0052066.s005.pdf]

Figure S6

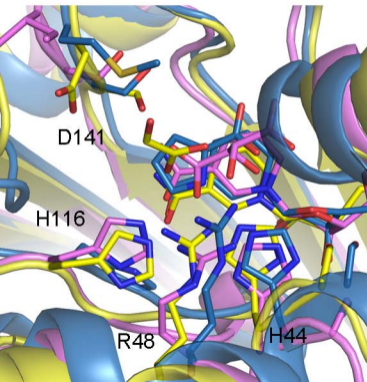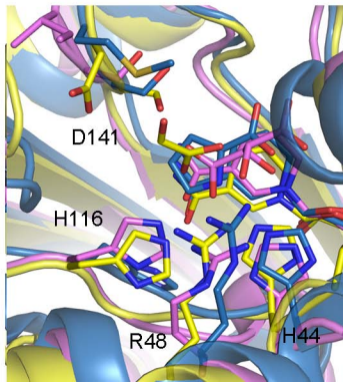

Supplement: Figure S6 — Binding site for ligand in AllD, YiaK, and DpkA. Ligand-binding sites are shown for the ternary complex of AllD with glyoxylate (yellow), YiaK with an inhibitor (magenta; PDB code 1S20) [25], and DpkA with a substrate analog (blue; PDB code 2CWH) [24]. It is noticeable that histidine residue corresponding to His116 in AllD is present in a structure of DpkA, but its orientation is quite different from that in AllD and YiaK. (PDF) [file pone.0052066.s006.pdf]

Figure S7

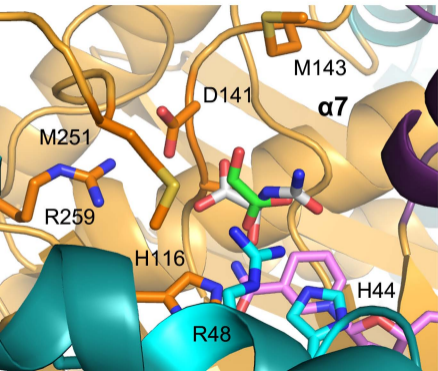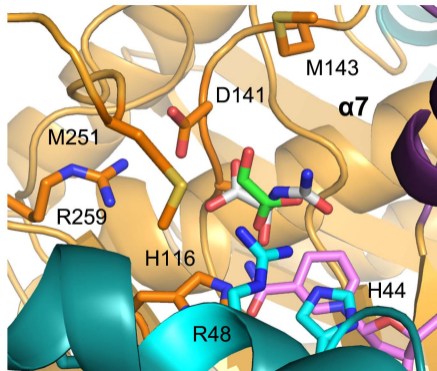

Supplement: Figure S7 — The proposed binding mode of (S)-ureidoglycolate. We modeled the binding mode of (S)-ureidoglycolate (gray), based on the functional analysis and a proposed mechanism. The coordinates are based on those of (S)-ureidoglycine [14], and after placed in the active site, the model was subject to an energy-minimization step in the program CNS [26]. For comparison, the model for glyoxylate (green) and NADH (magenta) in the ternary complex is indicated. Details for the interactions are described in the text. (PDF) [file pone.0052066.s007.pdf]

Figure S8

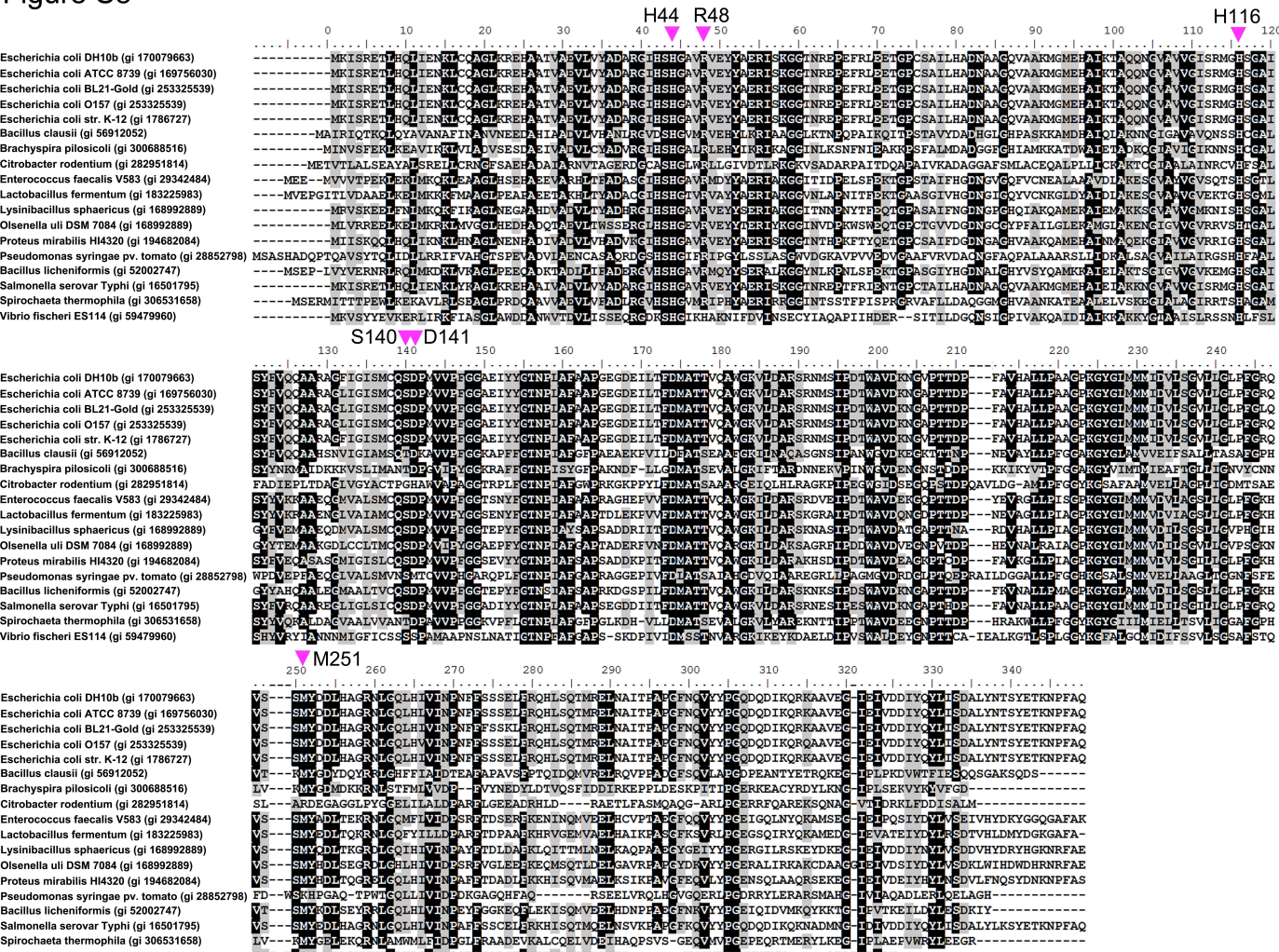

Supplement: Figure S8 — Sequence alignment of (S)-ureidoglycolate dehydrogenases annotated from various microorganisms. Gene access number is given in parentheses and several active site residues are indicated. (PDF) [file pone.0052066.s008.pdf]

Figure S9

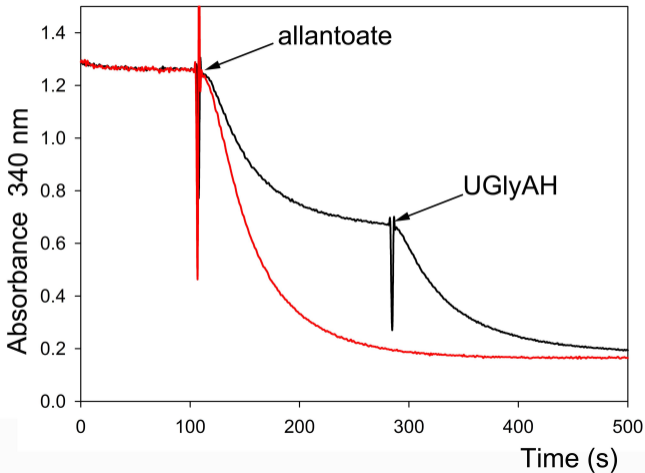

Supplement: Figure S9 — Formation of (S)-ureidoglycolate. This measurement was performed according to the procedures described previously [13], [14]. Accordingly, 2.5 mM α-ketoglutarate, 0.3 mM NADPH, 5 units of glutamate degydrogenase (Sigma-Aldrich), and AAH (2.07 µM; 105.2 µg/mL) were incubated, then 0.15 mM allantoate (Sigma-Aldrich) and UGlyAH (0.69 µM; 22.3 µg/mL) were subsequently added to the reaction mixture (shown in black). Absorbance decrease at 340 nm by addition of allantoate and UGlyAH is due to the released ammonia in each reaction, representing conversion of NADPH to NADP+. In an alternate experiment (red), UGlyAH was included in the pre-reaction mixture, followed by initiating the reaction with 0.15 mM allantoate. After approximately 3 min, the reaction was completed. Our calculation for the conversion of NADPH into NADP+, with a molar extinction coefficient of 6220 M−1 cm−1 at 340 nm for NADPH, indicated a complete conversion of allantoate into (S)-ureidoglycolate. (PDF) [file pone.0052066.s009.pdf]
