# Supplementary material for: Structural and Functional Insights into (S)-Ureidoglycolate Dehydrogenase, a Metabolic Branch Point Enzyme in Nitrogen Utilization
Source: PLoS One. 2012 Dec 20;7(12):e52066. doi: 10.1371/journal.pone.0052066 (PMC3527362; doi:10.1371/journal.pone.0052066)
Supplement: Table S1 — Primer sequences used in this study. (PDF) [file pone.0052066.s010.pdf]

Table S1. Primer sequences used in this study.

Proteins			Sequence
AAH		Forward primer	5’- GGAATTCCATATGATTACACATTTCCGTCAAG - 3’
		Reverse primer	5’- AACCGCTCGAGTTATTTCTGCCAGGCAAG -3’
UGlyAH		Forward primer	5’-GGAATTCCATATGGATGATGGGTTTTGTTCAG - 3’
		Reverse primer	5’-AACCGCTCGAGTCACAATGGATTTCGATTCAC -3’
AllD	WT	Forward primer	5’- GGAATTCCATATGAAAATCAGTCGGGAAACACTC - 3’
		Reverse primer	5’- AACCGCTCGAGTTATTGCGCAAAGGGATTTTTC -3’
AllD	S43A	Forward primer	5’- GCCAGAGGGATCCACGCTCATGGCGCGGTG CGC -3’
		Reverse primer	5’- GCGCACCGCGCCATGAGCGTGGATCCCTCTGGC -3’
AllD	H44A	Forward primer	5’- AGAGGGATCCACTCTGCTGGCGCGGTGCGCGTG - 3’
		Reverse primer	5’- CACGCGCACCGCGCCAGCAGAGTGGATCCCTCT - 3’
AllD	R48A	Forward primer	5’-TCTCATGGCGCGGTGGCCGTGGAATACTACGCG - 3’
		Reverse primer	5’- CGCGTAGTATTCCACGGCCACCGCGCCATGAGA - 3’
AllD	Y52F	Forward primer	5’- GTGCGCGTGGAATACTTCGCGGAACGCATTTCA - 3’
		Reverse primer	5’- TGAAATGCGTTCCGCGAAGTATTCCACGCGCAC - 3’
AllD	H116A	Forward primer	5’- ATCAGCCGGATGGGTGCCAGCGGCGCAATCTCT - 3’
		Reverse primer	5’-AGAGATTGCGCCGCTGGCACCCATCCGGCTGAT - 3’
AllD	S140A	Forward primer	5’- ATTTCGATGTGCCAGGCCGATCCAATGGTGGTG - 3’
		Reverse primer	5’- CACCACCATTGGATCGGCCTGGCACATCGAAAT - 3’
AllD	D141A	Forward primer	5’- TCGATGTGCCAGTCCGCTCCAATGGTGGTGCCG - 3’
		Reverse primer	5’- CGGCACCACCATTGGAGCGGACTGGCACATCGA - 3’
AllD	D141N	Forward primer	5’- TCGATGTGCCAGTCCAATCCAATGGTGGTGCCG - 3’
		Reverse primer	5’- CGGCACCACCATTGGATTGGACTGGCACATCGA - 3’
AllD	D141E	Forward primer	5’- TCGATGTGCCAGTCCGAACCAATGGTGGTGCCG - 3’
		Reverse primer	5’- CGGCACCACCATTGGTTCGGACTGGCACATCGA - 3’
AllD	M251A	Forward primer	5’- CTACAGGTTAGTTCGGCGTATGACGATTTACAC - 3’
		Reverse primer	5’- GTGTAAATCGTCATACGCCGAACTAACCTGTAG - 3’
AllD	R259A	Forward primer	5’- GATTTACACGCCGGGGCTAATTTGGGGCAATTA - 3’
		Reverse primer	5’- TAATTGCCCCAAATTAGCCCCGGCGTGTAAATC - 3’

Restriction sites used for cloning are underlined, and the boldfaced-underline shows the mutated sequences.
